# Subdural hematomas: glutaric aciduria type 1 or abusive head trauma? A systematic review

**DOI:** 10.1007/s12024-015-9698-0

**Published:** 2015-07-29

**Authors:** Marloes E. M. Vester, Rob A. C. Bilo, Wouter A. Karst, Joost G. Daams, Wilma L. J. M. Duijst, Rick R. van Rijn

**Affiliations:** Department of Radiology, Academic Medical Center, Room G1-213, Meibergdreef 9, 1105 AZ Amsterdam, The Netherlands; Department of Forensic Medicine, Netherlands Forensic Institute, The Hague, The Netherlands; Medical Library, Academic Medical Center, Amsterdam, The Netherlands; GGD IJsselland, Zwolle, The Netherlands

**Keywords:** Metabolic disorder, Glutaric aciduria type 1, Subdural hematoma, Abusive head trauma, Forensic radiology

## Abstract

**Purpose:**

Glutaric aciduria type 1 (GA1) is a rare metabolic disorder of glutaryl-CoA-dehydrogenase enzyme deficiency. Children with GA1 are reported to be predisposed to subdural hematoma (SDH) development due to stretching of cortical veins secondary to cerebral atrophy and expansion of CSF spaces. Therefore, GA1 testing is part of the routine work-up in abusive head trauma (AHT). This systematic review addresses the coexistence of GA1 and SDH and the validity of GA1 in the differential diagnosis of AHT.

**Methods:**

A systematic literature review, with language restriction, of papers published before 1 Jan 2015, was performed using Pubmed, PsychINFO, and Embase. Inclusion criteria were reported SDHs, hygromas or effusions in GA1 patients up to 18 years of age. Of 1599 publications, 20 publications were included for analysis.

**Results:**

In total 20 cases, 14 boys and 6 girls, were included. In eight cases (40 %) a child abuse work-up was performed, which was negative in all cases. Clinical history revealed the presence of trauma in eight cases (40 %). In only one case neuroradiology revealed no abnormalities related to GA1 according to the authors, although on evaluation we could not exclude AHT.

**Conclusion:**

From this systematic review we conclude that SDHs in 19/20 children with GA1 are accompanied by other brain abnormalities specific for GA1. One case with doubtful circumstances was the exception to this rule.

## Introduction

Subdural hematomas (SDHs) in children, especially under two years of age, are a common finding in abusive head trauma (AHT). This refers to inflicted cranial, cerebral, and spinal injuries due to blunt force trauma (e.g., acceleration or deceleration trauma), inertial trauma (e.g., repetitive acceleration–deceleration trauma), or a combination of factors [1, 2]. In contrast it has been reported that SDHs are estimated to be present, without significant trauma, in 20–30 % of children with glutaric aciduria type 1 (GA1) [3–8]. GA1 is a rare, autosomal recessive, metabolic disorder caused by a deficiency of riboflavin-dependent glutaryl-CoA dehydrogenase. Glutaryl-CoA dehydrogenase is a mitochondrial matrix enzyme which helps to convert the proteins lysine, hydroxylysine, and tryptophan to acetoacetyl-CoA. The deficiency of this enzyme results in accumulation of the neurotoxic breakdown products glutaric acid and 3-hydroxy-glutaric acid [9, 10]. The first patient description of GA1 was 1975, since then over 200 mutations have been described [11, 12]. The disorder is estimated to have a world-wide birth prevalence of 1 in 100,000 with new-born screening methods [13, 14]. Twenty-five percent of patients remain unaffected, independent of their genotype [15, 16]. The clinical presentation of GA1 is very variable, even within families [17]. Macrocephaly is present at birth in most cases or else it develops shortly after birth. Prior to the new-born screening program most children first were presented, between the ages of 3–36 months, due to an encephalic crisis [18–22]. This encephalic crisis is mostly seen after a febrile illness and may result in bilateral striatal necrosis with dystonia, orofacial dyskinesia, and choreathetosis with an initially preserved cognitive function [16, 23]. As GA1, if diagnosed and treated early, is considered to be a treatable disorder it is part of the new-born screening programs in various countries [24, 25].

**Fig. 1:**
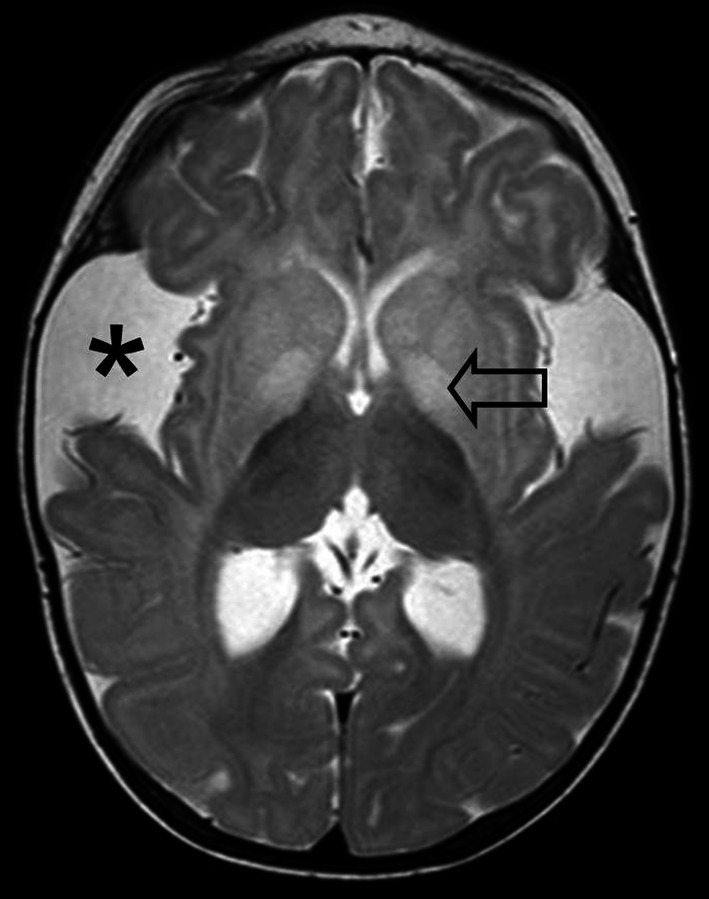
Six month old girl with known GA1. T2 weighted MRI (TR 3944, TE 80, Flip angle 90°, slice thickness 3 mm) shows widening of the Sylvian fissures (*asterisk*) and a high signal intensity of the basal ganglia (*arrow*)

Neuroimaging studies of GA1 patients are among others characterized by widening of the Sylvian fissures (open opercula), widened mesencephalic cisterns, expansion of CSF spaces anterior to the temporal lobes (Fig. 1) [4, 26]. These brain anomalies are the result of abnormal growth rather than atrophy and this has also been named micrencephalic macrocephaly [27]. In GA1 widening of the subarachnoid space can lead to tension on bridging veins which in turn are more susceptible to rupture, even after minor trauma, leading to SDHs. The presence of SDHs might be a diagnostic pitfall in the diagnosis of GA1, because SDHs can be misdiagnosed as the result of AHT. Based on this many pediatric guidelines recommend to consider GA1 in cases of SDHs due to suspected AHT [8, 25]. Diagnosis of GA1 might be life-saving and accordingly prevent false accusations and family disruption [16]. Nevertheless, from a clinic-forensic perspective it is important to note that diagnosis of GA1 does not exclude AHT [28].

The purpose of this study was to describe and analyze published SDH cases in children with GA1. A systematic review was performed to address the following research questions; is AHT considered and ruled out in case of a SDH in children with GA1? Was GA1 known at the time of presentation with SDH? Is there, based on the literature, a role for GA1 in the differential diagnosis of SDHs in AHT?

## Materials and methods

### Search strategy

A systematic literature search was performed in MEDLINE (Pubmed), EMBASE (OvidSP), PsychINFO (OvidSP), CINAHL, and the Cochrane library, for abstracts and articles up to 1 Jan 2015. It consisted of an indexed search with terms of glutaric aciduria type 1, non-accidental injury, child abuse, abusive head trauma, and subdural hematoma, performed by a literature search expert (JD). Publication language was restricted to English, German, French, and Dutch. Publication status was not restricted. In addition, citation tracking was performed in Google Scholar, Pubmed, and Web of Science. An overview of the search strategy can be given on request.

### Study selection

All identified articles, once scanned for duplicates, were screened by two researchers (R.R. and M.V.) for eligibility. In case of disagreement between the two researchers on either the evidence type of the article or whether the study met the inclusion criteria, a consensus was met after discussion. Titles were selected based on GA1 patient descriptions, a GA1 neurosurgical perspective or neuroimaging, and metabolic disorders combined with abuse, neuroimaging or SDHs. Selected abstracts were evaluated based on GA1 with SDHs, neuroimaging, neurosurgery or macrocephaly. After abstract selection, full articles were obtained and appraised. The references cited in the included full articles were manually examined for identification of additional relevant articles.

In the non-abused children ranking of exclusion of abuse was based on the Cardiff Child Protection Systematic Reviews [29]. This ranking system consists of the following categories: type A1 independently witnessed accidental cause or forensic recreation of scene; A2 by confirmation of organic disease (diagnostic test and/or diagnosis from clinical profile); B1 by multi-disciplinary assessment and child protection clinical investigation; B2 consistent account of accident by the same individual over time; B3 by checking either the child abuse register or records of previous abuse; C1 accidental cause/organic diagnosis stated but no detail given; C2 no attempt made to exclude abuse/no detail given. Studies were included if they had: (1) human study objects up to 18 years old, (2) study objects diagnosed with GA1, and (3) confirmed SDHs with CT and/or MRI scans. If studies did not relate which data belonged to which specific case, GA1 wasn’t confirmed, or no neuroimaging of SDH was available, they were excluded.

### Quality assessment

Based on criteria defined by the National Health Service’s Centre for Reviews and Dissemination and the CORE database, standardized data extraction and critical appraisal forms were used [29, 30]. Methodological quality assessment was performed by two individual researchers (R.R. and M.V.) using an adapted form of the Critical Appraisal Skills Program (CASP) (available upon request) [31].

### Data extraction

Data extraction was performed by one reviewer (M.V.), using a structured case report form (available upon request). The extracted data, at a patient level, were the following:

- Study design, patient characteristics (age, gender), author, year of publication, country, related family with GA1, and confirmed GA1 at time of SDH presentation.
- Type of imaging, other cerebral abnormalities at time of SDH presentation, clinical signs, and outcome.
- Consideration of AHT, steps taken to investigate this possibility (medically, legally, and socially) and investigation of SDH origin. Ranking of exclusion of child abuse according to CORE INFO [29].

## Results

### Study identification

**Fig. 2:**
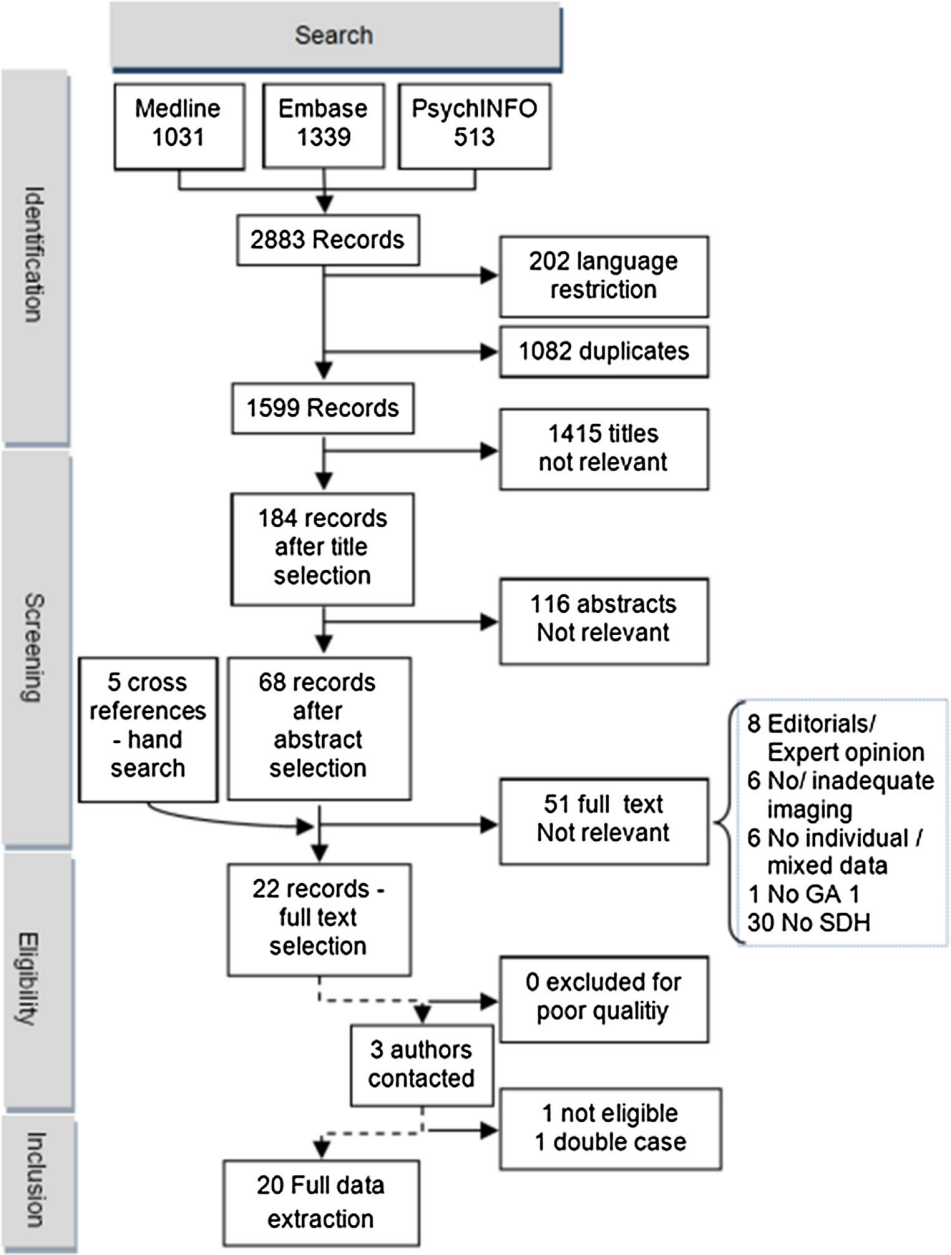
Flowchart literature search

The initial literature search yielded 1599 publications, after title and abstract selection 108 articles remained for full text evaluation (Fig. 2). Based on the analysis of the references cited in the included full texts we included five additional publications. However, none of these publications contained sufficient information for inclusion in our systematic review.

A total of 22 publications were selected, and three of the authors of these publications were contacted for additional information. One article was not eligible for inclusion because it contained insufficient information [27]. Furthermore, two articles described the same case, so only the article by Muntau et al. [7, 32] was included. Twenty articles were included in the final study, each of these containing 1 eligible patient description (Table 1) [3, 8, 16, 32–48]. This resulted in an inclusion of in total 20 children with GA1 and SDHs in this review.

**Table 1:** Included articles

Article	First author and ref. no.	Year	Journal	Country
1	Amir [33]	1989	J. Pediatr.	Denmark
2	Land [41]	1992	Neuropediatrics	UK
3	Osaka [43]	1993	Brain Dev.	Japan
4	Woelfe [8]	1996	Pediatr. Radiol.	Germany
5	Pfluger [44]	1997	Eur. Radiol.	Germany
6^a^	Muntau [32]	1997	Monatsschr. Kinderheilkd.	Germany
7	Lütcherath [42]	2000	Acta. Neurochir.	Norway
8	Hartley [16]	2001	Pediatrics	UK
9	Knapp [47]	2002	Pediatr. Emerg. Care	USA
10	Desai [35]	2003	Invest. Radiol.	USA
11	Gago [37]	2003	Retina	USA
12	Elsori [36]	2004	East. Mediterr. Health J.	Kuwait
13	Singh [46]	2006	Ind. J. Radiol. Imaging	India
14	Hou [38]	2007	J. Neurosurg.	USA
15	Bishop [3]	2007	J. Neurosurg.	USA
16	Kamate [39]	2009	Ind. J. Pediatr.	India
17	Carman [34]	2012	J. Pediatr. Child. Health	Turkey
18	Kim [40]	2014	An. Clin. Lab. Sci.	Korea
19	Zielonka [48]	2014	J. Child. Neurol.	Germany
20	Pusti [45]	2014	Case Rep. Pediatr.	India

^a^Same patient also described by Köhler et al. [7]

### Study quality

The 20 articles consisted of 4 case series and 16 case reports [34, 39]. Higher evidence levels than level 4 and 5 of case reports and case series were not available.

### Subdural hematoma’s in children with glutaric aciduria type 1

The included cases consisted of 14 boys (80 %) with a median age of 10 months (range 8 weeks–2 years) and 6 girls with a median age of 14 months (range 6–23 months) (Table 2). Of all cases only patient number 3 did not have an enlarged head circumference or macrocephaly defined as >2 SD. The head circumferences of child 1 and 17 were not reported.

**Table 2:** Clinical information of included cases

Pt^a^	Age^b^	Sex	Medical history and clinical signs	AHT	CORE score^c^	SDH treatment	Outcome
1	18 Months	F	1 Months GA1 diagnosis. Mild psychomotor retardation	NR	C2		Diet and medication up till 18 months Dystonic quadriplegia at 3-year old
2	19 Months	M	Macrocephaly. Head trauma (5–6 stairs); sleepy, vomiting +1 Day; alternating consciousness, loss of head control, impaired speech and abnormal arm extension	NR	A2	VP shunt	Right sided hemiparesis, hypotonia, choreoathetosis. +6 weeks CT; increase subdural collection. 24 Months; normal movement, tonus and speech improvement 25 Months; drain infection > dystonia > urine/serum GA1 positive (previous results negative) 33 Months; sudden death
3	6 Months	M	5 Months: infection > focal motor seizures, involuntary movements. 6 Months: lethargic, dystonic hypotonia, oral dyskinesia	No trauma or abuse reported	C2	Bilateral craniotomy	Urine/fibroblast = GA1 positive 10 Months; clinical improvement with extrapyramidal symptoms
4	6 Months	F	Macrocephaly > normal US. Loss of head control, moderate trunk hypotonia	No trauma	C2	Left sided drainage and VP shunt	9 Months; progressive motor loss and dystonia. MRI: rest SDH Urine/fibroblast: GA1 positive
5	11 Months	F	Macrocephaly. Unclear head trauma; unconscious for hours, somnolence, muscle hypotonia and seizures	Social, clinic and radiology misinterpreted as battered child syndrome	B1	Shunt	+3-Year; loss of psychomotor function, dystonic-dyskinesia syndrome +4-Year; reanalysis > positive GA1 analysis
6	8 Months	M	Macrocephaly, 6 months; truncal hypotonia. 8 Months; fall from stairs; vomiting, somnolence, irritability, skin hematoma	Ophthalmology; bilateral multiple pin-point hemorrhages, AHT with parents discussed yet excluded after normal skeletal survey	B1		At 3-year old no symptoms
7	1-Year	M	Macrocephaly, delayed motor development. Acute encephalitis-like syndrome	NR	C2	Craniotomy and VP shunt	Deterioration after operation 2.5 Year; GA1 diagnosed 3-Year; death after metabolic crisis
8	8 Weeks	M	Mother 25-year old; cognitive impaired; social services involved Head against the wall by brother; vomiting, somnolence, right sided seizures. Macrocephaly	X-scull; no fractures. Normal coagulation. Skeletal survey: fractures right radius and metaphysic distal radius + periosteal reaction. Ophthalmology: 1 pinpoint hemorrhage right fundus	B1	Drainage: blood	+2 Weeks; AHT charges made by the police > temporary foster care 6 Months; developmental delay, bilateral hearing loss > urine/fibroblast: GA1 positive Radius fracture was a vein, charges against mother suspended, however the child remained in foster care
9	9 Months	M	Fall backwards from kneeling; stiffness > hypotonia with perioral cyanosis 5–10 min, normal CT. +1 days; vomiting (ear infection), +3 days; vomiting, dehydration	Loss of body control, no bleeding/family disorders, relative macrocephaly Ophthalmology: multiple intra retinal + 1 subhyaloid hemorrhage + papilledema Skeletal survey; normal	B1	Craniotomy and subdural drain	+6 Weeks fall from furniture; stiffness, rhythmic seizures all extremities, 5 min unconscious, and vomiting. Serum/urine: GA1 positive Infection > subdural-peritoneal shunt. Eventually gastrostomy, seizures and severe dystonia > bilateral pallidotomy
10	9 Months	M	Cyanosis, diarrhea, transient left focal seizures, shoulder girdle weakness, macrocephaly	Ophthalmology: bilateral retinal hemorrhages, no AHT reported	C2		30 Months; mild choreoathetosis and psychomotor retardation
11	6 Months	M	Macrocephaly and developmental delay	Ophthalmology: bilateral intraretinal + right sided vitreous hemorrhages. Skeletal survey normal	B1	Drainage	Urine: GA1 positive 4 Months; improvement of symptoms
12	10 Months	M	Macrocephaly 5 Months; meningitis +1 Weeks fever, cough, vomiting. Short left sided seizures, later right sided +5 Days hemiplegia	NR	C2		Urine/fibroblast: GA1 positive 3.5 Year; no seizures and mild improvement hemiplegia
13	8 Months	M	Macrocephaly, motor delay, milestone regression, dystonia, dysarthria and dyskinesia	NR	C2		Urine: GA1 positive
14	9 Months	M	Macrocephaly.	Ophthalmology normal; AHT excluded Urine > GA1 positive	B1	Bilateral burr holes	+2 Months; new right subdural collection; complete resolution in time
15	7 Months	F	Macrocephaly, mild hypotonia and milestone delay	AHT suspicion, mother denies Ophthalmology and skeletal survey; no signs for AHT	B1	Bilateral subdural drains	11 Months; macrocephaly, improvement hypotonia and head control, normal milestone development
16	2-Year	M	‘Breath-holding spells’ and macrocephaly	NR	C2	Drainage	Increase of macrocephaly and hypotonia
17	17 Months	F	Fall from chair with single generalized seizure	NR	A2	Operation	24 Months; increase muscle tonus, loss of milestone development
18	16 Months	M	Multiple head trauma’s, macrocephaly. Complex febrile convulsions and developmental delay, mild axial hypotonia	NR	C2	Burr holes	New symptomatic infection episode 28 Months; normal development
19	23 Months	F	Macrocephaly. Head trauma (50 cm fall) > vomiting +2 Days generalized seizure, unconsciousness, coma, anisocoria, less pupil reflexes, respiratory insufficient	NR	A2	Left sided hemi-craniotomy and VP shunt	Spastic tetraparesis, bilateral dystonia, axial hypotonia and bilateral pes equinus +9 Months; improvement most symptoms except axial hypotonia and orofacial dyskinesia
20	3 Months	M	Progressive macrocephaly, insufficient head control, motoric developmental delay	Normal blood count, no AHT reported	C2		+3 Months normal head control

*NI* neuroimaging, *SDH* subdural hematoma, *GA1* glutaric aciduria type 1, *CT* computed tomography, *MRI* magnetic resonance imaging, *US* ultrasound, *CSF* cerebrospinal fluid

^a^Patient number correlates with article number in Table 1

^b^Age of patient at time of diagnosis of subdural hematoma

^c^Cardiff Child Protection Systematic Reviews, ranking of exclusion of abuse A1 independently witnessed accidental cause or forensic recreation of scene; A2 by confirmation of organic disease (diagnostic test and/or diagnosis from clinical profile); B1 by multi-disciplinary assessment and child protection clinical investigation; B2 consistent account of accident by the same individual over time; B3 by checking either the child abuse register or records of previous Abuse; C1 accidental cause/organic diagnosis stated but no detail given; C2 no attempt made to exclude abuse/no detail given [29]

Heredity was apparent in four cases; child 12 and 20 had consanguine parents. Moreover, patient 1 was born from consanguine parents and also had GA1 affected relatives. The disorder was diagnosed prenatally in child 6 because of a brother with GA1. The confirmation of a GA1 diagnosis was at a median age of 12 months for boys and 22 months for girls. In four cases (20 %) the disease was already confirmed prior to presentation with a SDH (1, 6, 9, and 19). One of these, child 19, was identified by new-born screening.

In 18 out of 20 cases (6 and 7) urine screening tests with elevated levels of glutaric acid and/or 3-hydroxyglutaric were reported (Table 2). Enzyme activity was reported to be absent in 5 cases, decreased in 2 and not reported in 13 (65 %). All children started medical and dietary treatment once the diagnosis GA1 was established. Surgical intervention for the SDH was performed in 13 children (65 %), of which four received a ventricular-peritoneal shunt, three a subdural shunt and six drainage through either craniotomy or burr holes.

### Abusive head trauma work-up

For eight cases (40 %) a child abuse work-up was performed, four had a social background check and/or clinical history investigation, two had coagulation tests, five underwent skeletal surveys (of which 1 was a skeletal scintigraphy) and seven children underwent ophthalmologic assessment. In none of these cases child abuse was confirmed, although child 8 remained in the care of social services. Clinical history revealed head trauma in eight cases (40 %), varying from falling while kneeling to falling off a flight of stairs. Eleven cases did not report any cause for SDH development and one case presented after a metabolic crisis due to infection. In conclusion, child abuse was excluded in seven cases with a rank B1, in three children with rank B2 and in ten children no attempt was made to exclude abuse or at least this was not reported in the case report (rank C2) [29].

### Neuroimaging of SDHs and GA1

SDH neuroimaging was performed with a CT-scan in 13 cases, 13 with an MRI and 1 with ultrasound. Abnormalities of the brain were visible in 19 cases (95 %), these abnormalities were fronto-parietal brain atrophy in 19 (95 %), open opercula in 18 (90 %), white matter abnormalities in 7 (35 %), arachnoid cysts in 1 (5 %), ventricular dilatation and/or widening CSF spaces in 8 (40 %), and basal ganglia attenuation in 9 (45 %) (Table 3).

**Table 3:** Neuroradiological findings in included cases

Pt no.^a^	FLA	OO	BG	CSF	WA	AC	SDH
1	+	+	−	+	+	−	+
2	+	+	−	+	−	−	+
3	+	+	−	−	−	−	+
4	+	+	+	+	+	−	+
5	+	+	−	+	−	−	+
6	+	+	−	+	−	−	+
7	+	+	−	−	−	+	+
8	+	NR	−	−	+	−	+
9	−	−	−	−	−	−	+
10	+	+	+	+	+	−	+
11	+	+	−	−	−	−	+
12	+	+	−	−	−	−	+
13	+	+	+	+	+	−	+
14	+	+	+	−	−	−	+
15	+	+	+	−	−	−	+
16	+	+	+	−	+	−	+
17	+	+	−	−	−	−	+
18	+	+	+	−	−	−	+
19^b^	+	+	−	−	−	−	+
20	+	+	+	+	+	−	+

*FLA* frontal lobe atrophy, *OO* open opercula, *BG* basal ganglia increased attenuation, *CSF* ventricular and/or subarachnoid space dilatation, *WA* white matter abnormalities, *AC* arachnoid cysts, *SDH* subdural hematoma, *NR* not reported

^a^Patient number correlates with article number in Table 1

^b^Visible after decompressive surgery

Only one case, presented in the case report by Knapp et al. [47], revealed no neuroradiological abnormalities in keeping with GA1. The authors present the case of a 9-month old boy who was seen after a backward fall while kneeling, resulting in brief stiffness followed by limpness for 5–10 min and perioral cyanosis. At the ER a CT-scan of the brain showed no abnormalities. A day later he started to vomit, at that time the ED physician diagnosed otitis media and he was sent home. Two days later he was admitted to the same community hospital because of vomiting and subsequent dehydration. At this time a CT revealed a right-sided isoattenuating parietal subdural hematoma. No intervention was performed and, as on the next morning he was doing well, he was sent home again. Six weeks later the boy reportedly fell off the furniture resulting in stiffness, rhythmic jerking of all extremities, 5 min loss of consciousness, irritability, and vomiting. On CT bilateral hypoattenuating extra axial fluid collections and a right acute subdural hemorrhage were seen for which a craniotomy with subdural drain placement was performed. He was transferred to a pediatric hospital where clinical history and physical examination revealed that the boy could not yet sit independently and had only recently started to crawl, he also showed mild hypotonia of the trunk, and relative macrocephaly. On ophthalmologic assessment there were multiple intraretinal hemorrhages, 1 subhyaloid hemorrhage and mild papilledema. There were no significant coagulation disorders or fractures on the skeletal survey. The child protection team advised GA1 testing, which was found to be positive. After interviewing the parents and careful evaluation, AHT diagnosis was rejected.

We contacted the authors of this case for additional information on the suspicion of child abuse and for supplementary CT-scan images. Unfortunately, patient information was de-identified for the authors and no additional information could be given. The case was reviewed by two forensic pediatric medical doctors (RB and WK) with respectively 27 and 7 years of experience in the field and a forensic pediatric radiologist (RR) with 12 years of experience. The clinical history, as reported, is insufficient from a forensic perspective. Ocular subhyaloid hemorrhages are not characteristic of GA1, it can be a result of migration hemorrhages due to retinal hemorrhages and/or retinoschisis, as can be seen in AHT [49, 50]. Furthermore the force of the initial impact, as described by the parents, is under normal circumstances insufficient to cause a SDH [51]. The predisposition in children with GA1 for the development of SDH is believed to be a result of stretching of cortical veins secondary to cerebral atrophy/hypoplasia and expansion of CSF spaces [38]. Conversely, it has been suggested that a metabolic crisis in GA1 children causes cerebrovascular changes such as arteriolar dilatation, increased cerebral blood volume, and consequently venous hypertension possibly leading to SDH development [52]. At both injury times only the parents were around and therefore no independent witnesses can testify to what happened. In this case, lack of metabolic crisis symptoms, absence of widened subarachnoid spaces and otherwise normal appearance of the first CT scan, does not lead to an increased risk for SDH development. From a forensic point of view we therefore conclude that in this specific case AHT could not be completely ruled out.

## Discussion

To our knowledge this is the first systematic review conducted on SDHs in children with the metabolic disorder of GA1. Twenty GA1 children with a SDH were identified in literature. All but the one case by Knapp et al. [47] were accompanied by other neuroimaging abnormalities specific for GA1. In 19 out of 20 cases cerebral imaging showed brain abnormalities specific for GA1. MRI and CT of the brain in these cases show widening of insular cisterns (Sylvian fissure/open opercula) in 93 % of patients in the literature and 90 % in this review [35, 53, 54]. These specific findings should therefore always lead to further investigation of GA1 [35]. Other distinctive cerebral changes reported in GA1 children with SDHs are comparable to reported GA1 cases without SDHs. Commonly this comprises of bilateral frontotemporal cortical atrophy or hypoplasia and sometimes enlargement of ventricles and mesencephalic cisterns [26, 35]. In 180 GA1 patients evaluated by Osaka et al. [43] abnormalities of the putamen, caudate nucleus, cortex, ventricles, and external CSF spaces were more often found on MRI in children with severe movement disorders. Notably changes of the putamen and enlarged ventricles are clinical important predictors of the severity of the disease [55]. Most of these abnormalities are detected during or after a metabolic crisis. Moreover on MRI these white matter abnormalities can improve after aggressive therapy [56].

Although in young children AHT is one of the main causes of SDH formation, there certainly is a differential diagnosis. Among others it has been recognized that in children with benign enlargement of the subarachnoid space (BESS) there is an increased risk of developing SDHs, after even a minor trauma, and that thus SHDs are not pathognomonic of AHT in these children [57]. A large retrospective study evaluated a total of 177 children with BESS; in this population 4 (2.3 %) were diagnosed with a SDH [58]. All children were evaluated for suspected AHT and in one case healing rib fractures were diagnosed, this patient was subsequently reported to child protective services. This implies that even with a potential benign cause for the presence of a SDH child abuse should be considered. The mechanism, i.e., stretching of anchor veins, as seen in BESS is also present in GA1 and is the cause of an increased risk for SDH development [59]. We found that in only 40 % of included cases AHT was considered and actively eliminated. Acute symptoms of SDHs, e.g., vomiting were the main reason for GA1 diagnosis in 60 % of reported cases. It is worrisome that in 60 % of cases no work-up for child abuse was presented in the case reports, we can only assume that no work-up was performed. It is important to note that the presence of GA1 does not exclude the possibility of abuse as a matter of fact children with metabolic disorders, as are all children with chronic diseases, might be even more vulnerable to AHT [3, 28, 60–63].

The strength of this systematic review is impaired by the fact that most included references were case reports and some case series. However, other study types are unlikely to be performed in this rare patient group. Due to the language restriction relevant articles might have been missed however it is a common approach when performing a literature search to exclude languages. Another issue is the variance in the use of terminology in medical literature. In 2009 the AAP has recommended to use the term AHT instead of shaken baby syndrome [2]. As this recommendation does not apply to older literature many different terms are in use, which potentially may lead to cases being missed in the literature search. Also in the field of radiology there are inconsistencies in terminology, terms such as acute subdural hematoma, chronic subdural hematoma, and subdural effusions are used interchangeably [64, 65].

## Conclusion

From this systematic review we conclude that in 19 out 20 cases SDHs in children with GA1 are accompanied by other brain abnormalities specific for GA1. Based on our findings we feel that the time has come to remove GA1, in case of otherwise normal neuroimaging confirmed by a radiologist, as one of the standard differential diagnoses of SDHs in AHT suspicion.

## Key points

1. The relatively high 20–30 % estimated incidence of subdural hematomas in children with GA1 is probably due to frontal lobe atrophy and other brain abnormalities resulting from high levels of neurotoxic intermediate breakdown products.
2. We found a total of 20 cases of subdural hematomas in children with GA1 which have been published since the discovery of this disease in 1975.
3. 19 out of 20 children in this series with GA1 and SDH had brain pathology which could predispose to later subdural hematoma development. In the one exception abusive head trauma was thought to be possible.
4. There is no supporting evidence for a role of GA1 in medical differential diagnosis of AHT in case of an otherwise normal CT or MRI scan of the brain.
